# *Ascaris lumbricoides* infection: Still a threat for iron deficiency anaemia in 2-year-old Bangladeshi slum-dwelling children

**DOI:** 10.3855/jidc.11340

**Published:** 2019-10-31

**Authors:** Md. Shabab Hossain, Subhasish Das, Md. Amran Gazi, Mustafa Mahfuz, Tahmeed Ahmed

**Affiliations:** 1 Nutrition and Clinical Services Division, International Centre for Diarrheal Disease Research, Bangladesh (icddr,b), Dhaka, Bangladesh; 2 James P. Grant School of Public Health, BRAC University, Dhaka, Bangladesh; 3 Department of Global Health, University of Washington, Seattle, WA, United States

**Keywords:** Anaemia, *Ascaris lumbricoides*, Bangladesh, children, iron deficiency, parasitic infection

## Abstract

**Introduction:**

Although parasitic infections lead to extracorporeal iron loss resulting in iron deficiency anaemia (IDA), data associating IDA with parasitic infections in the first two years of life are limited. We sought to evaluate the prevalence and severity of anaemia and IDA during this period and to investigate the association between intestinal parasitic infections and IDA.

**Methodology:**

Data was collected under MAL-ED study protocol in Bauniabadh slum of Dhaka, Bangladesh. The presence of parasites in stool was detected using wet preparation microscopy at 7, 15, and 24 months. Anaemia was defined as serum haemoglobin < 11 g/dL and IDA was defined by serum haemoglobin < 11 g/dL, serum ferritin < 12 g/L and soluble transferrin receptor > 8.3 mg/L. Logistic regression was done to quantify the relation between stool parasite and IDA separately on samples collected at 7, 15 and 24 months.

**Results:**

265 children were enrolled after birth and samples were collected at 7, 15 and 24 months. Anaemia was detected at 7, 15 and 24 months in 117 (48.8%), 106 (44.2%) and 67 (27.9%) cases whereas IDA was found in 15 (6.3%), 47 (19.6%) and 39 (16.3%) cases, respectively. Iron deficiency anaemia at 24 months was significantly associated with *Ascaris lumbricoides* infection (OR 3.76; 95 % CI, 1.08-13.11).

**Conclusions:**

The prevalence of anaemia and IDA in slum dwelling children of Dhaka is high and Ascaris lumbricoides infection was found to have a strong association with IDA at 24 months of age.

## Introduction

Anaemia is a major public health problem worldwide, with an estimated global prevalence of 24.8% [1]. The World Health Organization (WHO) has classified anaemia as a severe public health problem (prevalence *≥* 40%) for children under the age of five [2]. This burden of anaemia is mostly borne by the developing world, affecting as many as two-thirds of children under the age of five living in Africa and South East Asia [1]. The scenario is much worse in the comparatively younger children of the South-Asian region, affecting roughly 70-80% children under two years of age [3]. According to recent information, the prevalence of anaemia among children under the age of three was about 79 % in India [4]. And above 50% of these anaemia cases are due to iron deficiency (ID) [5]. Most national surveys do not specifically measure markers of iron status along with haemoglobin [3], resulting in a knowledge gap in assessing iron deficiency. According to the World Health Organization (WHO), iron from breast milk is not enough to meet child’s iron requirement beyond the age of six months [6]. The amount of iron in breast milk is highest in the first month, but it decreases gradually in the subsequent periods and is reduced down to 0.3 mg/L approximately at the fifth month [7]. According to the WHO, 98% of the iron requirement in infants aged 6–23 months should be met by solid foods [8]. If infants are fed with iron-poor foods after the sixth month, which is quite common in the urban slums of Bangladesh, they exhaust almost all of their iron stores and iron deficiency develops [9].

The report of Nutritional Surveillance Project (NSP) of Helen Keller International (HKI) in 2001 shows that the prevalence of anaemia in children of pre-school age in Bangladesh was 48% [10]. Although the National Micronutrient Status Survey 2011-2012 (NMSS 2011-12) reported this prevalence to have reduced to 33% [11], the prevalence of anaemia in children under two years of age in a nationally representative survey was still found to be as high as 49.0% (H Rashid; Nagoya J Med Sci, 2009) [11]. The underlying causes of anaemia are multi-factorial, though there is no data available that depicts the contribution of the associated factors resulting in anaemia [10]. However, as already mentioned, 50% of anaemia cases are due to iron deficiency according to global estimates, a condition known as iron deficiency anaemia (IDA) [5]. Iron deficiency, the most common single micronutrient deficiency worldwide [12], affects 30% of the world’s total population [13] and more than 50% of children in developing countries [14]. As consequences of IDA, infants and young children may suffer from reduced immune function, poor growth and irreversible cognition and motor function deficits [15-17]. The prevalence of ID and IDA in children of pre-school age in Bangladesh is 10.7% and 7.2%, respectively [11], but is unknown for two-year old children. The principal causes of IDA are parasitic infections and low iron intake [2]. Infectious diseases, particularly parasitic infections lead to extracorporeal iron loss which increases iron requirements as well as the risk of IDA [2,18].

Intestinal parasitic infections are among the most common infections worldwide, significantly affecting children [19]. It is noted that both protozoan and helminthic infections are very common among children under the age of five [20]. Studies show that intestinal parasitic infections are present all over Bangladesh throughout the year [18]. In Bangladesh, the most common infective parasites include *Entamoeba histolytica*, *Giardia lamblia*, *Ascaris lumbricoides, Trichuris trichura*, hookworms and *Enterobius vermicularis* [18]. These infections directly and passively result in various public health problems and can cause nutritional impairment and anaemia, and retard physical and mental development [18].

Because of the knowledge gap relating the association of parasitic infection with IDA in the first two years of life, we sought to evaluate the prevalence of anaemia as well as IDA in Bangladeshi two-year-old children and to determine the association of specific intestinal parasites with IDA.

## Methodology

### Study site and data collection

The study was conducted under The Etiology, Risk Factors and Interactions of Enteric Infections and Malnutrition and the Consequences for Child Health and Development (MAL-ED) study protocol in the urban slum of Bauniabadh, Mirpur-11, Dhaka, Bangladesh [21]. In the birth cohort component of MAL-ED study, 265 healthy newborn were enrolled within the first 17 days of life. The enrolment took place from February 2010 to February 2012. After obtaining ethics committee approval and written informed consent from the parents and taking into account the drop-outs from the study due to refusal to consent and migration from the study area, stool and blood samples were collected from each participant at 7, 15 and 24 months of age, resulting in 629 and 577 samples, respectively. At each time point, out of 265 stool samples, 224, 207 and 198 samples could be collected at 7, 15 and 24 months, respectively. And out of 265 blood samples, 207, 195 and 175 samples could be collected at 7, 15 and 24 months, respectively. Possibly due to the invasive nature of blood sample collection, the number of blood samples was lower than the stool samples at each time point. The MAL-ED study had a well-defined recruitment protocol with very stringent inclusion and exclusion criteria [21]. For instance, mothers were asked before enrolment whether the family had plans to move outside the community. This included planned absence from the study area of > 30 days, which would have made it difficult for the field-worker to contact the mother or caregiver during that time. Exclusion criteria for cohort recruitment were maternal age of < 16 years, not a singleton pregnancy, another child already enrolled in the MAL-ED study, severe disease requiring hospitalisation prior to recruitment, and severe acute or chronic conditions diagnosed by a physician (e.g., neonatal disease, renal disease, chronic heart failure, liver disease, cystic fibrosis, congenital conditions) [22].

### Laboratory analyses

All laboratory analyses were performed in the laboratories at icddr,b in Dhaka, Bangladesh. The presence or absence of parasites in stool was detected using wet preparation stool microscopy except for *Giardia lamblia, Entamoeba histolytica* and *Cryptosporidium*, which were detected by using commercial Enzyme Linked Immuno Sorbent Assay (ELISA) kit. Iron deficiency anaemia was defined by serum haemoglobin (hb%) level < 11 g/dL, serum ferritin level < 12 μg/L and soluble transferrin receptor (sTfR) > 8.3 mg/L [14]. Serum ferritin was adjusted for inflammation by C-reactive protein (CRP) and Alpha-1-acid glycoprotein (AGP).

### Statistical analyses

Statistical analyses were performed using IBM SPSS Statistics for Windows version 20.0 (IBM Corporation, New York, NY, USA). Mean values, standard deviation (SD) and 95% confidence intervals (CI) of means were used to describe the distribution and prevalence. Apart from observing the prevalence of anaemia, IDA and parasitic infection in children, another vital aim of the study was to compare the status and progress of each of these events within this two-year timeline. Keeping this in mind, this time period was further divided at three points to obtain comparative view of the events. So, logistic regression analysis was performed to quantify the relation between stool parasite status and iron deficiency anaemia separately on samples collected at 7, 15 and 24 months separately.

### Results

Overall, 265 children aged 0-24 months were enrolled in the study and 240 were available for analysis.50.8% of study participants were female. Table 1 describes the median values of Hb%, ferritin and sTfR values at 7, 15 and 24 months. The median hb% level was found to be normal at 24 months, but was below the normal level at 7 and 15 months. At 7 months the median ferritin level was higher than normal and that of 15 and 24 months. The median sTfR values were normal at each time-point.

**Table 1:** Descriptive characteristics of the participants at 7, 15, and 24 months.

Variables	7 months	15 months	24 months
Gender, n (%)			
Male	118 (49.2%)		
Female	122 (50.8%)	-	-
Hb%* (g/dL), median (IQR)	10.7 (10.1-11.4)	10.4 (9.5-11.3)	11.2 (10.1-12.1)
CRP* (mg/L)†	0.95 (0.40, 2.90)	0.60 (0.30, 2.20)	0.60 (0.15, 1.70)
median (IQR)			
AGP* (mg/dL)†	89.00 (68.75, 115.25)	86.00 (69.00, 109.00)	72.00 (55.00, 95.00)
median (IQR)			
Ferritin (μg/L),	30.50 (16.00, 48.80)	12.80 (6.83, 21.80)	8.00 (4.80, 14.50)
median (IQR)			
Ferritin (μg/L),	25.9 (12.8-42.4)	10.7 (5.7-18.1)	7.2 (4.7-13.9)
median (IQR)‡			
sTfR* (mg/L), median (IQR)	5.2 (4.4-6.8)	7.2 (5.2-9.6)	6.9 (5.3-10.8)

* AGP = alpha-1-acid glycoprotein; CRP = C-reactive protein; hb% = serum haemoglobin; sTfR = soluble transferrin receptor; IQR = Inter-quartile range

† Normal ranges: CRP, ≤ 10 mg/L; AGP, ≤ 100 mg/dL

‡ Adjusted for elevated CRP and elevated AGP by mathematical correction.

**Figure 1:**
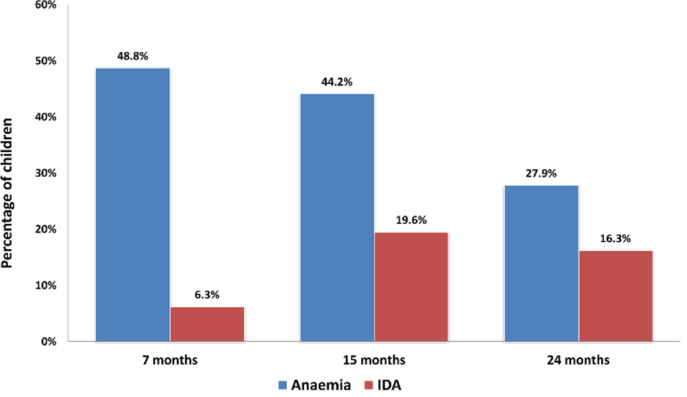
Prevalence of anaemia and IDA at 7, 15 and 24 months.

Figure 1 shows the prevalence of anaemia and iron deficiency anaemia at 7, 15 and 24 months of age. The incidence of anaemia decreased as the time progressed. On the other hand, the incidence of IDA significantly went up about 3 fold at 15 months and sustained afterwards.

**Figure 2:**
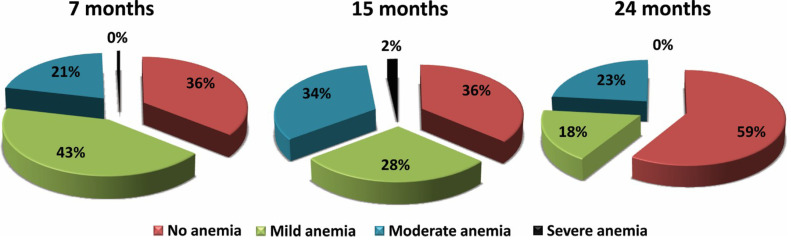
Graphical representation of anaemia by severity. Results: 265 children were enrolled after birth and samples were collected at 7, 15 and 24 months. Anaemia was detected at 7, 15 and 24 months in 117 (48.8%), 106 (44.2%) and 67 (27.9%) cases whereas IDA was found in 15 (6.3%), 47 (19.6%) and 39 (16.3%) cases, respectively. Iron deficiency anaemia at 24 months was significantly associated with Ascaris lumbricoides infection (OR 3.76; 95 % CI, 1.08-13.11).

Figure 2 shows the percentage distribution of severity of anaemia among children studied by age group as per the WHO guidelines [23]. According to the guidelines for this particular age group, no anaemia, mild anaemia, moderate anaemia and severe anaemia was defined as hb% levels > 11.0 g/dL, 10.0-10.9 g/dL, 7.0-9.9 g/dL and < 7.0 g/dL, respectively. The results indicate that the prevalence of mild anaemia declined gradually as time progressed. Moderate anaemia, however, persistently remained above 20% throughout the whole timeline.

**Figure 3:**
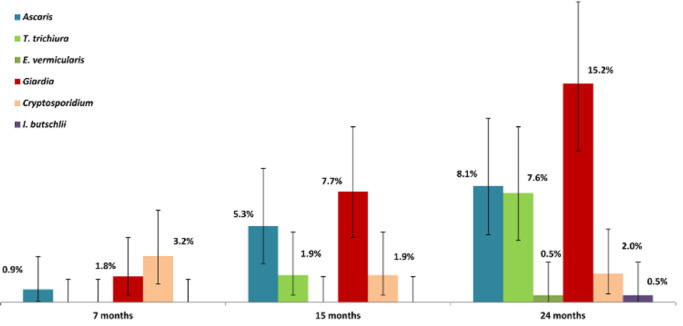
Prevalence of intestinal parasitic infection at 7, 15 and 24 months. Conclusions: The prevalence of anaemia and IDA in slum dwelling children of Dhaka is high and Ascaris lumbricoides infection was found to have a strong association with IDA at 24 months of age.

Figure 3 shows the prevalence of intestinal parasitic infection at 7, 15 and 24 months of age. The most prevalent intestinal parasite at this age group was *Giardia intestinalis*, followed by *Ascaris lumbricoides* and *Trichuris trichiura*. *Cryptosporidium*, *Enterobius vermicularis* and *Iodamoeba butschlii* were detected in a small proportion of children.

Table 2 shows the association of certain parasites with iron deficiency anaemia at different time points. Among which, the only significant association was observed in the case of *Ascaris lumbricoides* at 24 months. Iron deficiency anaemia at 24 months was significantly associated with ascariasis (OR 3.76; 95 % CI, 1.08-13.11). On the other hand, due to the low prevalence of all parasites at 7 months, as well as a total absence of *Trichuris trichiura,* no association was observed between any parasites and IDA at this particular time point. The prevalence of certain important parasites including *Entamoeba histolytica*, *Enterobius vermicularis* and hookworms were found to be extremely low at each time point. As a result, those parasites were not included in the regression model.

**Table 2:** Association of parasites with IDA at different time points

Parasite	Time point	OR (95% CI)	P-value
*Ascaris lumbricoides*	7 months	0 (0.00,0.00)	0.999
	15 months	1.03 (0.25,4.21)	0.969
	24 months	3.76 (1.08,13.11)	0.038
	7 months	-	-
*Trichuris trichiura*	15 months	0 (0.00,0.00)	0.999
	**24 months**	**0.71 (0.17,2.99)**	**0.645**
	7 months	0 (0.00,0.00)	0.999
*Cryptosporidium*	15 months	0.80 (0.08,7.96)	0.849
	24 months	0.67 (0.05,8.28)	0.755
	7 months	6.08 (0.51,72.03)	0.152
*Giardia intestinalis*	15 months	1.50 (0.46,4.90)	0.502
	24 months	1.64 (0.59,4.51)	0.342

OR = Odds ratio; CI = Confidence interval.

## Discussion

Our study revealed a high prevalence of anaemia during the first two years of life of slum-dwelling children. This results show similarity with the national data, where the prevalence of anaemia among slum-dwelling children aged 6-23 months was 45% [11]. Therefore, anaemia still remains a significant public health problem. Although we have the IFA supplementation program for pregnant women, there is no anaemia control program for children. Specific interventions and control programs are needed to prevent and control anaemia in children, as well.

In our study, we found that the incidence of anaemia decreased with age. This is also similar to the national level data, where the prevalence of anaemia was 49% in children aged 6-23 months and 32% in children aged 24-59 months [11]. Studies show that anaemia tends to reduce with age [4]. The prevalence of iron deficiency anaemia spiked initially from 7 to 15 months then showed a gradual decline at the end of 24 months. Severe anaemia was not found at 24 months of age and at a very insignificant rate at 7 or 15 months as well. However, the prevalence of mild and moderate anaemia throughout the whole timeline was significant. This could be due to a universal breast feeding habit at this age. According to the WHO, iron from breast milk is not enough to meet a child’s iron requirement beyond the age of 6 months [6]. The amount of iron in breast milk is highest in the first month, but gradually decreases in the subsequent periods and is reduced down to approximately 0.3 mg/L at the fifth month [7]. According to the WHO, 98% of the iron requirement in infants aged 6–23 months should be met by solid foods [8]. If infants are fed with iron-poor foods after the sixth month when they exhaust almost all of their iron stores, iron deficiency develops [9]. This is possibly the case in participants of this study, as well.

Though the incidence of anaemia was highest, the prevalence ofIDA was found to be lowest at 7 months of age. This leaves the question of the cause of anaemia in these non-iron deficient children. Different micronutrient studies have shown that children of this age are deficient in other vital micronutrients such as zinc, folic acid, vitamin B6, B12 and retinol apart from iron [3,24,25]. This might have a possible impact in anaemia, as well. Further studies investigating the relation between other micronutrients and anaemia are necessary to test this correlation.

The results of this analysis show a high prevalence of intestinal parasitic infections among the participants. The most prevalent intestinal parasite was *Giardia intestinalis*, followed by *Ascaris lumbricoides* and *Trichuris trichiura*. However, studies show that intense infection with intestinal parasites is less common in children under the age of five compared to five-year old children and older [6]. The high prevalence of intestinal parasites may be attributed to poor hygiene, which enables the parasites to complete their life cycles between the environment and humans. Despite the high coverage of national deworming programs, this data highlights the importance of regular screening for intestinal parasites in children of the mentioned age group.

No hookworm infestations were identified in our study, which is consistent with results obtained in other studies [19,26,27] conducted in urban localities but inconsistent with the results of a study conducted in rural areas [28]. This shows that the parasitic profile of urban areas is not quite similar to that in rural areas. A meta-analysis shows that footwear use is associated with lower odds of hookworm infestation [29]. The incidence of intestinal parasitic infection increased with age. This could be due to the fact that as the child grows older the exposure to soil and many of the other risk factors for intestinal parasitic infection increase [19].

Iron deficiency anaemia at 24 months was significantly associated with *Ascaris lumbricoides* infection. Certain parasites, especially soil-transmitted helminths (STHs), live in the intestine and feed on the blood of the host tissue leading to the loss of iron and protein [30]. *Ascaris lumbricoides*, an STH that lives in the intestine, sucks blood and damages the intestinal wall, causes blood leakage which results in anaemia. They receive nutrition from sucking the blood from the gut of affected persons and lay thousands of eggs, which get passed through the feces of infected persons and matures into a form that is infective [31]. In areas of inadequate sanitation and with open-air defecation practices, it contaminates the soil and easily gets transmitted to children living in that area by ingestion of the eggs. This may occur when hands or fingers with contaminated dirt on them are put in the mouth or by consuming vegetables or fruits that have not been carefully cooked, washed or peeled [32].

## Conclusion

The prevalence of anaemia as well as iron deficiency anaemia in Bauniabadh slum, Mirpur, Dhaka is high and *Ascaris lumbricoides* infection was found to have a strong association with IDA at 24 months of age. Specific interventions and control programs are needed to prevent and control anaemia as well as iron deficiency anaemia and regular screening for intestinal parasites is warranted for children of the mentioned age-group.
